# A New Approach for Gastrointestinal Tract Findings Detection and Classification: Deep Learning-Based Hybrid Stacking Ensemble Models

**DOI:** 10.3390/diagnostics13040720

**Published:** 2023-02-14

**Authors:** Esra Sivari, Erkan Bostanci, Mehmet Serdar Guzel, Koray Acici, Tunc Asuroglu, Tulin Ercelebi Ayyildiz

**Affiliations:** 1 Department of Computer Engineering, Cankiri Karatekin University, Cankiri 18100, Turkey; 2 Department of Computer Engineering, Ankara University, Ankara 06830, Turkey; 3 Department of Artificial Intelligence and Data Engineering, Ankara University, Ankara 06830, Turkey; 4 Faculty of Medicine and Health Technology, Tampere University, 33720 Tampere, Finland; 5 Department of Computer Engineering, Baskent University, Ankara 06790, Turkey

**Keywords:** deep learning, stacking ensemble learning, gastrointestinal tract classification, endoscopy images, McNemar’s test

## Abstract

Endoscopic procedures for diagnosing gastrointestinal tract findings depend on specialist experience and inter-observer variability. This variability can cause minor lesions to be missed and prevent early diagnosis. In this study, deep learning-based hybrid stacking ensemble modeling has been proposed for detecting and classifying gastrointestinal system findings, aiming at early diagnosis with high accuracy and sensitive measurements and saving workload to help the specialist and objectivity in endoscopic diagnosis. In the first level of the proposed bi-level stacking ensemble approach, predictions are obtained by applying 5-fold cross-validation to three new CNN models. A machine learning classifier selected at the second level is trained according to the obtained predictions, and the final classification result is reached. The performances of the stacking models were compared with the performances of the deep learning models, and McNemar’s statistical test was applied to support the results. According to the experimental results, stacking ensemble models performed with a significant difference with 98.42% ACC and 98.19% MCC in the KvasirV2 dataset and 98.53% ACC and 98.39% MCC in the HyperKvasir dataset. This study is the first to offer a new learning-oriented approach that efficiently evaluates CNN features and provides objective and reliable results with statistical testing compared to state-of-the-art studies on the subject. The proposed approach improves the performance of deep learning models and outperforms the state-of-the-art studies in the literature.

## 1. Introduction

The gastrointestinal (GI) tract is a tubular system whose main task is digestion and includes many organs between the mouth and anus. The tubular structure of the GI tract consists of the mouth, pharynx, esophagus, stomach, small intestines, large intestines, rectum, and anal canal. The tubular structure is associated with all the salivary glands, gall bladder, liver, and pancreas organs. The tubular structure of the GI tract from the inside out is mucosa, submucosa, muscularis propria, and serosa. Most of the diseases of the GI tract occur with the deterioration of the innermost layer, the mucosa. Both benign and malignant diseases can occur in the GI tract, such as gastric, colon, small intestine, and rectum cancer, i.e., from GI tract cancers to malignant diseases; peptic ulcers, hemorrhoids, and celiac disease are examples of benign diseases. In addition to these diseases, inflammatory diseases such as Crohn’s disease and ulcerative colitis are also present.

In a study conducted by Sung et al. [1], according to Global Cancer Statistics 2020, it was estimated that 9.9 million of the 19.3 million new cancer cases resulted in death worldwide, regardless of gender. Of the approximately 10 million deaths, 9.4% are caused by colorectum, 7.7% by stomach, and 5.5% by esophageal cancer. The mortality rate of other GI tract diseases besides cancer is also remarkable. According to the report of the burden of digestive diseases in the Americas Region published by the Pan American Health Organization (PAHO) in 2021 [2], digestive diseases such as peptic ulcer, appendicitis, gastritis and duodenitis, and inflammatory bowel disease caused 375,170 deaths in 2019. In addition to these statistics, clinical studies prove that early detection of GI tract diseases is crucial to reducing mortality [3,4]. Early diagnosis is possible by detecting and distinguishing small polyps, adenomas, or lesions formed in the tubular structure.

Endoscopy is a device with a camera at the end that directly displays the inside of the organs. A gastroenterologist uses endoscopic methods such as gastroscopy and colonoscopy to diagnose pathological findings such as wounds, polyps, or tumors in the stomach, large intestine, or esophagus. At the same time, the treatments of hemorrhages and structures such as polyp lesions are performed with endoscopic methods. The risks that may occur during endoscopic procedures are extremely low, except for temporary side effects. However, endoscopic observations are dependent on specialist experience and variables between observers. Repetition of endoscopy is costly for patients who change hospitals or doctors. It is very laborious and time-consuming to examine past endoscopy videos of patients by any specialist and to provide the diagnosis by the specialist.

Computer-aided diagnosis (CAD) systems are a research area that aims to save time for the specialist in evaluating medical images and assisting the specialist in image interpretation as a second opinion. Although CAD systems have been researched for many years, their interest has recently increased with the advancement of artificial intelligence and medical imaging technologies. Especially with deep learning methods, very successful performance results were obtained in CAD studies, and diseases that specialists could not diagnose were visualized [5,6,7]. Although CAD-supported endoscopy devices are not standard in gastroenterology, several artificial intelligence-supported endoscopy devices that automatically detect diseases such as colon tumors, colon polyps, and colorectal cancer have been approved by the U.S. Food and Drug Administration and European Union [8].

There are many studies in which statistical analyses of specialist and artificial intelligence diagnoses related to gastroenterology are performed and prove that an artificial intelligence application can diagnose as well as an endoscopist [9,10]. Wang et al. [11] compared the effects of standard colonoscopy and a deep learning-based CAD system on polyp diagnosis. The authors reported that the CAD system detected more small adenomas (*p* < 0.001) compared to standard colonoscopy, while there was no statistical difference in larger adenomas (*p* = 0.075). The results of this study are promising that artificial intelligence applications can be more efficient than specialists in endoscopic diagnosis. An endoscopic examination specialist for diagnosing GI tract diseases decides by examining the spatial differences in the mucosal surface and tissue. The specialist may miss small polyps or lesions during this review and decision-making process. Making the diagnosis by a hybrid artificial intelligence model with high accuracy and sensitive measurements can offer many opportunities to help the specialist, such as cost, time, workload savings, early diagnosis, and objectivity in endoscopic diagnosis.

In general, the remaining parts of this article are as follows. Section 2 presents a literature review on the subject, the literature gaps, and the study’s contribution to the literature. After giving information about the background and datasets in Section 3, the architecture of the proposed approach applied, hyperparameters, and training details are presented. In Section 4, after giving information about the performance metrics selected for the testing phase of the proposed approach, the test results and statistical analysis are intertwined with the discussion. In Section 5, the article is concluded.

## 2. Related Works

Computer-aided gastroenterology diagnosis has been studied for more than 30 years, with datasets created using various endoscopic imaging methods. Diagnosis of abnormal pathological findings in only a specific part of the GI tract, especially polyp detection [12,13,14], is the most researched subject [15,16,17]. Studies covering the entire GI tract, such as pathological findings, anatomical signs, and therapeutic interventions detection and classification, have also taken their place in the literature [18]. In the first 20 years of this process, image processing techniques were used for feature extraction, and statistical methods were used for classification [19]. Features used to classify images can be divided into three categories: features in the spatial domain, features in the frequency domain, and features that describe images at a higher level. Pixel-based and histogram algorithms extracted features in the spatial domain, Fourier and wavelet transform algorithms extracted features in the frequency domain, and edge and region-based algorithms extracted high-level features. These features were generally classified using statistical machine learning methods.

With the rapid development of deep learning methods in the last decade, CNN (convolutional neural network) architecture, which enables the extraction and classification of spatial and high-level features, has been the focus of researchers. Hybrid methods using CNN features, transfer learning, designing new CNN models, and various deep learning networks are among the proposed methods [20]. Since CAD studies in the field of gastroenterology have a long history and a wide range of topics, this section includes in detail the studies that classify the KvasirV2 and HayperKvasir datasets that we used in our study.

The Kvasir dataset was presented to researchers in 2017 by Pogorelov et al. [21]. The first version of the Kvasir dataset contained 500 samples in each of the 8 classes, later updated to KvasirV2, and the number of samples doubled. Pogorelov et al. [21] performed the first multi-class detection experiment on Kvasir and achieved 95.00% ACC with three-layer CNN. The HyperKvasir dataset, an updated version of the KvasirV2 dataset containing high-quality samples, was introduced by Borgli et al. [22]. The authors trained the labeled data using several cutting-edge pre-trained CNNs and achieved a maximum MCC score of 90.20%. When the literature is examined, HyperKvasir still needs to be a researched dataset as Kvasir. Possible reasons for this are that the HyperKvasir dataset has a highly imbalanced sample distribution and is relatively new compared to the Kvasir dataset.

In recent studies, transfer learning has often been used to classify the KvasirV2 dataset. A study by Dheir and Abu-Naser [23] used VGG, ResNet, MobileNet, Inception-v3, and Xception networks, while in another study conducted by Hmoud Al-Adhaileh et al. [24], GoogleNet, ResNet-50, and AlexNet were used. The authors obtained classification results by freezing the convolution bases of pre-trained CNN models on the ImageNet dataset. The highest performance in Dheir and Abu-Naser’s study [23] was the VGG model, with 98.30% ACC. In comparison, the AlexNet model had the highest performance with 97.00% ACC in the study of Hmoud Al-Adhaileh et al. [24]. In the same way, Yogapriya et al. [25] used VGG16, ResNet-18, and GoogLeNet, and the VGG16 achieved 96.33% ACC. When the experimental results of studies using transfer learning are examined, the success of the VGG16 architecture in GI tract classification is remarkable.

Some researchers aimed to improve the performance of transfer learning methods by designing new classifiers on the convolution bases of pre-trained CNN models. Öztürk and Özkaya [26] used a new long short-term memory (LSTM)-based classifier for the classification layer of AlexNet, GoogleNet, and ResNet architectures. The LSTM-based CNN models were trained on the ImageNet dataset and then retrained on the KvasirV2 dataset as pre-trained models. Moreover, 97.90% ACC was obtained with the method proposed by the authors. The same authors [27] then extended their research with the residual LSTM layer CNN, which transmits features from each pooling layer to the LSTM layer, and increased the performance to 98.05%. Designing a new network by properly revising a known model according to the problem has often been presented as a solution in computer vision research. Dutta et al. [28] classified HyperKvasir dataset with 60 fps speed and 75.80% MCC with Tiny Darknet model seven times smaller than Darknet model’s size.

Research has focused on features obtained from endoscopic images, offering more feature extracting, selective, and combining algorithms than classifier designs. Ramamurthy et al. [29] presented a multi-feature fusion method for GI tract classification on the HyperKvasir dataset. In this method, images were classified by combining the features from pre-trained EfficientNetB0 and a special CNN named Effimix, and 97.99% ACC was achieved. Few GI tract disease detection and classification studies have focused on obtaining optimal deep-learning features. Using Bayesian optimization, Khan et al. [30] trained segmented lesion regions using transfer learning and initialized fine-tuned MobileNet-V2 hyperparameters. The authors achieved 98.02% ACC due to their experiments on the KvasirV2 dataset. In another study by Khan et al. [31], a method based on moth-crow optimization with distance-canonical correlation (D-CCA) fusion was proposed, and an ACC of 97.20% was obtained.

Mohapatra et al. [32] classified GI tract abnormalities using a method combining two-dimensional discrete wavelet transform (2D-DWT) and CNN architecture on the KvasirV2 dataset, achieving 97.25% ACC. Mohapatra et al. [33] classified the HyperKvasir set into two stages for the same task. In the first stage, images are divided into normal and abnormal classes, while in the second stage, they are subclassified. The authors used empirical wavelet transform (EWT) to extract specific patterns in the images and CNN for the two-step classification task. Moreover, 96.65% ACC was achieved in the first stage classification and 94.25% in the second stage classification.

Capsule networks are among the proposed methods for classifying the KvasirV2 dataset. In a study diagnosing GI tract diseases by selecting five classes from the eight-class KvasirV2 dataset, Afriyie et al. [34] used denoising capsule networks (Dn-CapsNets) and achieved 94.16% ACC. Wang et al. [35] proposed a two-stage classification method combining CNN and capsule networks to diagnose GI tract diseases automatically. The method for measuring performance on KvasirV2 and HyperKvasir datasets focuses on extracting lesion-aware CNN features. Although this feature provides an essential advantage for diagnosing GI tract diseases, it gives a limited performance in differentiating lesion-free classes of the GI tract. The authors reported a classification ACC of 94.83% in the KvasirV2 dataset and 85.99% in the HyperKvasir dataset with the convolutional-capsule network.

### 2.1. Literature Gaps

When the literature studies are examined, it is observed that most studies focus on diagnosing lesions, polyps, or diseases. CAD systems that can thoroughly examine the GI tract are needed to develop a system that can be used in clinical applications that assist specialists. Instead of focusing only on pathological findings, we present a study that can simultaneously detect and classify pathological findings, anatomical landmarks, therapeutic interventions, and mucosal views to meet this need. In addition to narrowing the research area in the literature, it is clear that the datasets studied are also limited. Instead of only one dataset, our study used KvasirV2 and HyperKvasir datasets, which contain different classes with balanced and unbalanced sample distributions. Working on two different datasets provides the opportunity to observe the behavior of the proposed method on datasets with balanced and unbalanced sample distribution by testing the performance of the proposed method on an ample sample space.

Another shortcoming of the studies in the literature is that no study has measured the statistical significance of the proposed methods. Even if the proposed algorithms give different performance values, the difference should be statistically significant. Artificial intelligence methods developed for medical applications must provide evidence for statistical significance, as in the medical literature. In our study, the statistical significance of each algorithm whose performance was measured among other algorithms was measured using McNemar’s test, and the selected performance criteria were supported by statistical analysis. The fact that our study thoroughly examines the GI tract without limiting the subject, tests the proposed approach on two different datasets, and performs statistical analyses indicates that we present more objective and transparent research than the studies in the literature.

It is observed that the methods proposed in the literature for the detection and classification of GI tract findings generally divide the problem into two feature extraction and classification, and the algorithms are shaped according to the side that the authors consider essential. Although feature fusion or selection algorithms successfully detect lesions or pathological findings in a particular region, they limit distinguishing samples from non-lesional tissues while classifying all GI tract findings. Studies focusing on classification algorithms designed to be used with CNN features in the literature have achieved higher performance than feature-oriented studies. An endoscopist decides on the diagnosis by examining textural differences in the image, similar to a CNN algorithm’s operation. CNN features represent spatial and high-level features. Although it is a disadvantage that time-dependent frequency features cannot be extracted with CNN filters, the CNN features obtained by learning to optimize the filters are sufficient to distinguish textural differences. To solve such a problem, the extraction of time-dependent frequency features increases the model complexity and computational cost, creates manual applications that work offline, and requires human intervention. In addition, the approaches available in the literature need to give better performance results to be used in clinical applications.

This study proposes a deep learning-based hybrid stacking ensemble approach for detecting and classifying GI tract findings. Our models, designed according to the stacking ensemble learning approach, consist of two levels. In the first level, predictions are obtained by applying 5-fold cross-validation to three new CNN models. In the second level, a selected machine learning classifier is trained according to the obtained predictions, and the final classification result is reached. The primary feature that makes the proposed approach powerful is that it has a hybrid decision-making mechanism that uses the prediction of more than one model by combining deep learning and machine learning approaches.

### 2.2. Contributions

The contributions of this study to the literature are listed below:

1. It is the most detailed and large-scale study in the literature. It can simultaneously detect and classify pathological findings, anatomical points, therapeutic interventions, and mucosal images on two datasets with balanced and unbalanced sample distribution.
2. It presents three new CNN models with high performance and low hyperparameter sensitivity to the literature.
3. An innovative hybrid approach to learning that efficiently evaluates CNN features and enhances deep learning models’ performance is proposed.
4. It is the first study in the literature to provide reliable and objective results in which performance results are supported by applying statistical tests other than metrics. This study is a good precedent for the statistical analysis of artificial intelligence methods.
5. The performance of the proposed approach is higher than other state-of-the-art methods proposed in the literature.

## 3. Materials and Methods

### 3.1. Proposed Approach

Ensemble learning is a meta-learning method that enables different algorithms to work collaboratively to reduce variance and increase bias and prediction performance [36]. Stacking is an ensemble learning method combining heterogeneous core learners with a meta-learner to output a predictive prediction and improve performance [37]. Generally, two-level stacking models are used, but stacking with multiple levels is possible. Different base learners at the first level make different assumptions and produce predictions. The meta-learner at the second level uses the predictions from the first level as features and combines the predictions to produce the final predictions.

**Figure 1:**
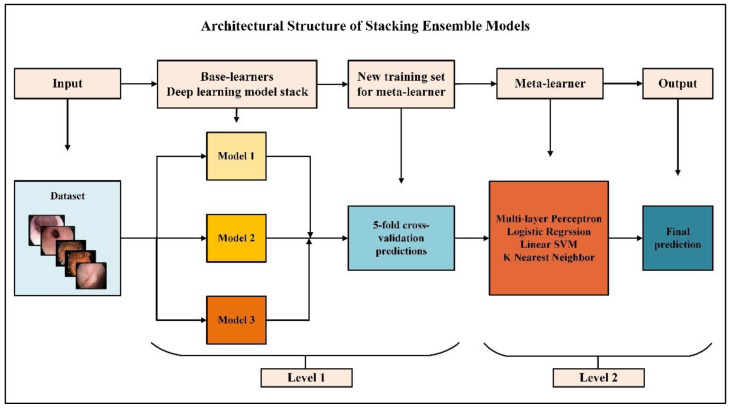
Overview of the proposed approach.

An overview of the proposed approach is presented in Figure 1. In the proposed approach, four different stacking models with two levels are established. Each stacking model consists of three identical heterogeneous base learners and a different meta-learner. Three different CNN models based on VGG16 architecture are designed for base learners. Training, validation, and testing phases were carried out using the same hyperparameters for the stacking models. The factor that distinguishes stacking models from each other is meta-learner selection. Logistic regression (LR), linear support vector machine (LSVM), multi-layer perceptron (MLP), and K-nearest neighbor (KNN) algorithms were chosen as meta-learners. In this article, three different CNNs designed as base learners are named Model 1, Model 2, and Model 3. Meta-learner hybridized with Model 1, Model 2, and Model 3 in which MLP is Stacking Ensemble Model 1 (SEM 1), LR is Stacking Ensemble Model 2 (SEM 2), LSVM is Stacking Ensemble Model 3 (SEM 3), and KNN is Stacking Ensemble Model 4 (SEM 4). The meta-learner must be a simple model with little complexity, as it is more likely to fit predictions from base learners. For this reason, less complex machine learning algorithms were chosen instead of deep learning algorithms as meta-learners. The stacking technique’s purpose is to increase base learners’ prediction performance. Given that each base model contributes the same amount to the ensemble prediction, the performance of each base learner must be high for the overall prediction performance to increase. Using CNNs with simultaneous feature extraction and classification for images as base learners is very suitable for architecture and performance.

The algorithm steps by which we implement the proposed stacking ensemble models are listed below:

1. The dataset was split into 5 using the stratified shuffle split cross-validation strategy;
2. Three independent base learners are trained to the other folds, keeping one of the folds, and predictions are obtained;
3. The above three steps were repeated five times to obtain out-of-sample predictions for all five folds;
4. All out-of-sample predictions were used as training data for meta-learners;
5. The final output was estimated with meta-learners.

**Figure 2:**
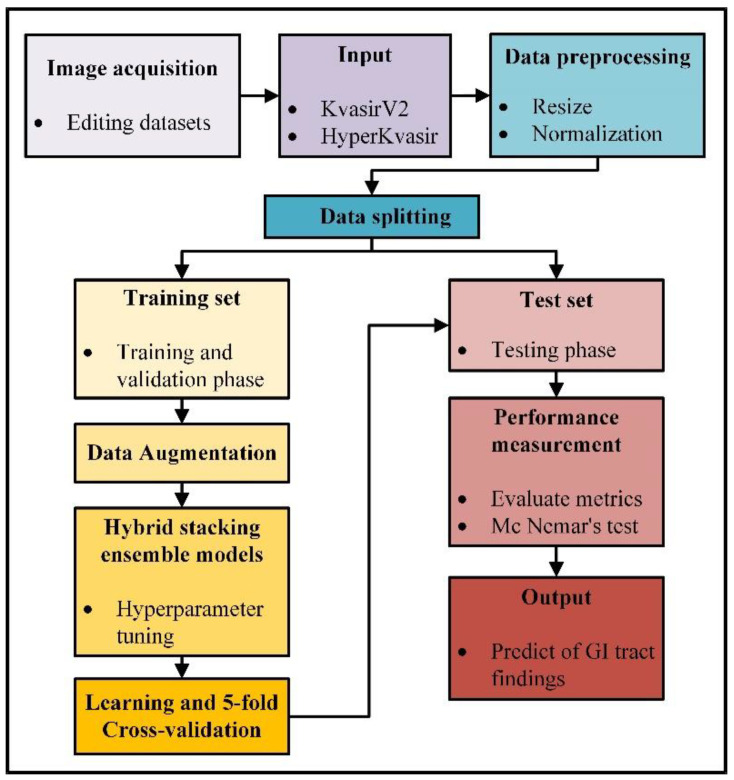
Overview of the experimental study steps.

An overview of the experimental study steps is presented in Figure 2. In the first step of the experimental study, KvasirV2 and HyperKvasir datasets were rearranged, and data pre-processing techniques were applied. Real-time data augmentation, model setup, hyperparameter tuning steps, and 5-fold cross-validation were applied to the training set, and training and validation phases were completed for each model. Results were obtained according to the performance metrics selected during the test phase, and the statistical analysis phase was carried out to evaluate the results.

### 3.2. Background

CNNs applied to analyze visual images are feed-forward neural networks that contain convolution operation instead of matrix multiplication in at least one of its layers. A CNN architecture is divided into the convolution base, which takes on the feature extraction task, and the classification base, which undertakes the classification task. In the convolutional base, feature maps are obtained by the sequential outputs of convolution, activation, and pooling layers. The raw image data coming from the input layer to the convolution layer are 3D tensors whose dimensions are width, height, and depth (channel), and these tensors are called feature maps. According to the two-dimensional convolution operation given in Equation (1), the product values obtained by multiplying each value in the feature map (I) with the value of the kernel (K) it matches are summed and thus converted into vector form. The 3D tensor output, namely the activation map (A), is created by bringing all these vectors side by side.

(1) $$A\left[m,n\right]=\left(I\times K\right)\left[m,n\right]=\sum_{j=-\infty }^{\infty }\sum_{i=-\infty }^{\infty }K\left[i,j\right]I\left[m-i,n-j\right]$$

Kernel size, padding, and stride are hyperparameters that affect the size of the activation map. Large kernel size selection is costly, so 3 × 3, 5 × 5, and 7 × 7 are CNN architectures’ most commonly preferred kernel sizes. For the input size to be equal to the output size, the padding (P) process is applied by adding rows and columns from all directions to the input. In the most commonly used zero-padding operation, zero is added to the input as rows and columns from all directions. The number of times the kernel is shifted on the attribute map is called the stride (S). Increasing the stride means decreasing the output. The size of the output of the convolution layer, whose width and height of the input image are equal, stride is taken as one or more, and padding process is performed, is calculated as given in Equation (2).

(2) A=(I−K+2P)/S+1

The activation layer determines which neuron will be active with a non-linear transformation. After the convolution layer, one of the ReLU, GELU, ELU, and Leaky ReLU activation functions is generally used [38]. After the fully connected layer, the sigmoid and softmax activation functions are used for the classification task. The task of the pooling layer is to reduce the following layer’s input image to reduce the network’s cost and prevent overfitting. The pooling filter, which slides over the image by the stride, combines the largest for maximum pooling, the average for average pooling, and the minimum for minimum pooling. The depth of the image emerging from the pooling layer does not change. The output size of the pooling layer is calculated as given in Equation (3). Fully connected layers are similar to classical multi-layer neural networks, and their task is to generate predictive output for classes.

(3) PO=(I−K)/S+1

Although CNN architecture is similar to MLP [39], a feed-forward artificial neural network, MLP loses its efficiency on high-dimensional data due to its complete interconnection. The difference between CNNs and MLPs is that CNNs have three-dimensional neurons, local connections, and common weights. 

The LR algorithm [40] uses the sigmoid function to classify non-linearly separable samples and generates predictions based on whether they are above or below a threshold. When a general regression model (Equation (4)) is used with the sigmoid function, the probability value (Equation (5)) for the output is calculated. The sigmoid function must go through the logarithmic transformation to calculate the error function (Equation (6)).

(4) y=α+βx

(5) $$p\left(x\right)=1/\left(1+e^{-\left(\alpha +\beta x\right)}\right)$$

(6) $$E\left(x\right)=\frac{1}{m}\sum_{i=1}^{m}\left(y_{i}-1\right)\log\left(1-p\left(x_{i}\right)\right)-y_{i}\log\left(p\left(x_{i}\right)\right)$$

The SVM algorithm [41] uses an optimal hyperplane to classify data. Support vectors are the data points closest to the hyperplane in the dataset. The vertical distances between the hyperplane and the support vectors are the margins. The SVM algorithm aims to find the optimum hyperplane to separate data points with different class labels and maximize margins. In order to prevent the margins from shrinking, the slack variable (ξ) is used, which allows some samples to be classified incorrectly. Learning of SVMs, including training data and labels ($x_{i}$, $y_{i}$) and parameters *w*, consists of the constrained optimization given in Equation (7). If the hyperparameter C used in Equation (7) is given small, misclassified samples are tolerated. The linear kernel is used if the data can be linearly separated in the input space.

(7) $$\frac{1}{2}‹w,w›+C\sum_{i=1}^{m}\xi_{i},\;y_{i}\left(‹w,x_{i}›+\beta \right)\geq 1-\xi_{i}\;,\;\xi_{i}\geq 0,\;i=1,2,\ldots ,m$$

The KNN algorithm [42] uses a distance measurement method to include the sample whose class is unknown in the closest class according to its distance from the other samples. The distance measurement method and the number of K neighbors are hyperparameters that affect performance. The label of most of the K-nearest neighbors determines the class of the unknown sample. In the Minkowski metric given in Equation (8), *p* = 1 is equivalent to the Manhattan metric, and if *p* = 2, it is equivalent to the Euclidean metric.

(8) $$d\left(x_{i},y_{i}\right)={\left(\sum_{i=1}^{m}{\left|x_{i}-y_{i}\right|}^{p}\right)}^{1/p},\;i=1,2,\ldots ,\;m$$

### 3.3. Datasets

**Figure 3:**
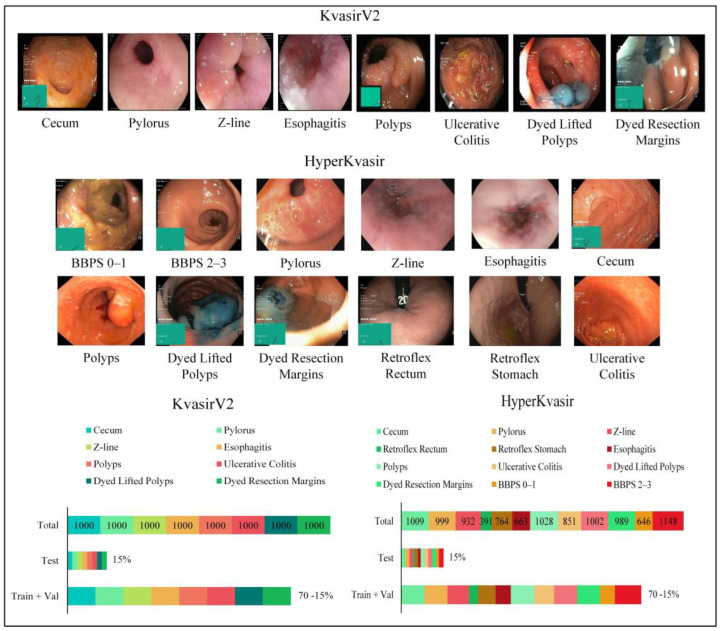
Sample images for each class from the datasets and the number of samples per class.

The Kvasir dataset has been tagged and validated by experienced endoscopists for the medical multi-media competition presented by MediaEval. The first version contains 4000 images, while the second version, KvasirV2, includes 8 classes and 1000 images per class, showing anatomical points in the GI tract (3 classes), pathological findings (3 classes), and therapeutic interventions (2 classes). Images are in different resolutions, from 720 × 576 pixels to 1920 × 1072 pixels. The HyperKvasir dataset was collected during examinations at Bærum Hospital in Norway and labeled by experienced endoscopists. The relatively large dataset is an expanded version of the Kvasir dataset and contains 110,079 images and 374 videos. Only 10,662 of these images are labeled and consist of a total of 23 classes showing anatomical points in the GI tract (6 classes), pathological findings (12 classes), therapeutic interventions (2 classes), and mucosal image quality (3 classes). The number of samples per class is uneven. Some classes, such as hemorrhoids (6 images) and terminal ileum (9 images), have insufficient samples for use in artificial intelligence applications. Ulcerative colitis and esophagitis classes are graded according to disease severity, and the number of samples for graded subclasses is insufficient. For example, the ulcerative colitis 1–2 class contains only 11 images. Classes with insufficient samples and subclasses of graded diseases in the HyperKvasir dataset were not used for the training phase to be efficient. In this study, all classes of the KvasirV2 dataset containing 8000 images were used. For the reasons stated, an arrangement has been made for the HyperKvasir dataset, and 12 classes containing 10,422 images have been used. The classes and image examples used for the application are presented in Figure 3.

Anatomical landmarks are reference points to explain the location of a particular finding. The Z line is the transition zone between the esophagus and the stomach and helps identify pathology in the esophagus. The pylorus is the area around the opening from the stomach to the duodenum and helps identify signs such as ulceration, erosion, or stenosis. The cecum is a region close to the large intestine that marks the colonoscopy completion. Retroflex rectum is the retroflection of the endoscope in the rectum to detect diseases in the rectal outlet. The retroflex stomach is the endoscope’s retroflection to visualize the stomach’s upper parts. The quality of mucosal images was classified according to the Boston Bowel Preparation Scale (BBPS) for complete visualization of the mucosa. Bowel cleansing is considered sufficient if the BBPS score is 2 or 3. Pathological findings are abnormalities in the mucosa due to the disease. Esophagitis is an inflammation of the esophagus, generally caused by conditions in which stomach acid flows back into the esophagus as reflux or vomiting. Polyps, which are protruding lesions in the mucosa, have the potential to develop into colorectal cancer. Ulcerative colitis is a chronic inflammatory disease of the large intestine. Therapeutic interventions such as polyp removal are performed during endoscopy. According to the endoscopic mucosal resection (EMR) technique, the dyed lifted polyps class shows the removed polyps, and the dyed resection margins class shows the resection site after polyp removal.

### 3.4. Details of Proposed Approach

Experimental studies were carried out in three stages: training, validation, and testing. Training, validation, and test sets were created using a 70-15-15% data split ratio and the stratified shuffle split method. The stratified shuffle split method is also one of the cross-validation methods. This method selects a determined ratio of samples from each class, and the problem of class bias that may occur in the validation and test set is prevented. The fact that all three datasets contain the same proportion of data from each class provides a more efficient and objective performance measure than the frequently preferred random split method. Several image pre-processing techniques were applied to all images as the working principle of CNN models. Pixel values have been normalized by rescaling to the 0–1 range. Due to the high-resolution input size increasing the training cost and the hardware used, all images were resized at 150 × 150, which is the appropriate size. The resizing process was performed with the bilinear interpolation algorithm. Color is an essential factor in distinguishing the disease in color endoscopic images. Therefore, all images were used in RGB (red, green, blue) mode, thus adding three channels to the input size (150 × 150 × 3). Adding artificial data obtained with data augmentation techniques applied to the original data to the training set increases the training performance of the network and prevents overfitting. With real-time data augmentation, 0.2 random rotation and width and height random zoom were applied to the training set. Points outside the boundaries of the input are filled with the nearest mode.

New CNN models based on the VGG16 [43] architecture were created for the first level of stacking models. There are several important reasons why the VGG16 architecture is based. The most important of these reasons is that it produced high-performance results in studies in which endoscopic images were classified using VGG16 architecture, according to the literature review. However, the features of the stacking ensemble learning models adopted in the proposed approach are compatible with the VGG16 modeling. The weak classifier of the base learners used for the first level is an important principle that reduces the model’s complexity. Compared to other CNN architectures, VGG16 has an architecture with less complexity and fewer parameters but with high classification performance.

The first two layers of the original VGG architecture have a 64-channel 3 × 3 filter. Next comes the maximum pooling layer with two strides. The next convolution layer uses a 256-channel 3 × 3 filter. After that, there are two sets, three convolution layers, and a maximum pooling layer. Each convolution layer has 512-channel filters of 3 × 3 size, with the same padding, and the image, after these steps, then passes into the stack of two convolution layers. The convolution layers contain the ReLU activation function. A batch normalization layer was added to the end of the convolution layers of the proposed CNN models, and Gaussian error linear unit (GELU) [44] was used as the activation function. The convolution and pooling layers are the same as the VGG16 architecture. The deep learning models’ configurations are presented in Table 1.

CNN processes data by dividing it into mini batches instead of processing them all at once. The data normalization process is the zero-centered and zero-to-one revaluation of the input data. The batch normalization layer applies a transformation, keeping the output standard deviation close to 1 and the average output close to 0. The fact that the normalization process can be optimized during training makes the model less sensitive to hyperparameter tuning. The low hyperparameter sensitivity allows a significant learning rate and lowers the importance of weight initialization. Since the mean and variances remain moderately constant across the entire network, the generalization error is reduced. The batch normalization layer is added before the convolution and activation layers to ensure that the network always produces activations with the desired distribution when updating a parameter value. The GELU function enables conversion between stochastic modifiers such as batch normalization or dropout layers and activation layers. This transformation is stochastic but depends on the value of the input. The ReLU [45] function multiplies by 0 if the input value is negative and by 1 if it is positive. GELU multiplies the input by a value between 0 and 1, determined by its input value. In Equation (9), the GELU activation function is expressed mathematically. Φ(x) is the cumulative distribution function of the standard normal distribution, and as P(X ≤ x) gets smaller, x decreases, so the GELU is more likely to multiply a neuron by 0. Since the neuron inputs follow a normal distribution when batch normalization is used, the GELU is chosen as the activation function in all convolution layers. MLP blocks consisting of dense, dropout, and batch normalization layers are used in fully connected layers that undertake the classification task. Model 1 includes one MLP block, Model 2 includes two MLP blocks, and Model 3 includes three MLP blocks.

(9) GELU(x)=xP(X≤x)=xΦ(x), Φ(x)=P(X≤x), X~N(0,1)

Application codes are written in Python using Keras [46] from deep learning libraries and Scikit-learn [47] from machine learning libraries. Hyperparameters of all models were selected using the distributed hyperparameter search strategy. Distributed hyperparameter search creates models by combining multiple specified hyperparameter values and running them in parallel. Thus, the combination of hyperparameters with optimum performance is determined. Distributed hyperparameter search was performed with Keras Tuner from the Keras library and GridSearchCV from the Sklearn library. The selected hyperparameters and their values are given in Table 2. The same hyperparameters are used in the base learners.

**Figure 4:**
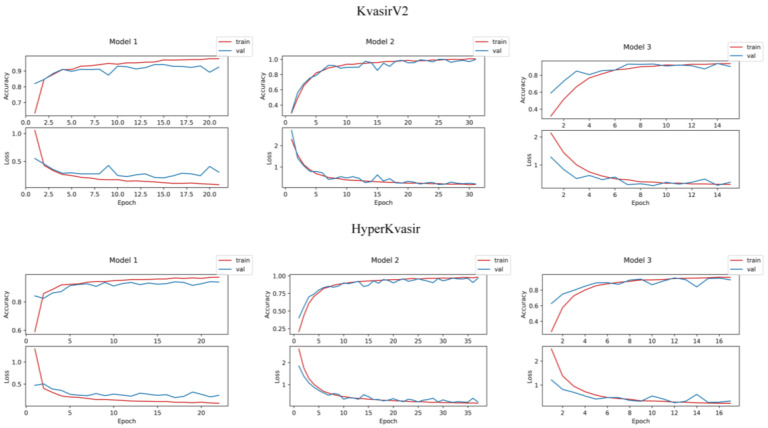
Learning curves of deep learning models.

Training, validation, and testing experiments were repeated on Model 1, Model 2, and Model 3 in order to compare the performances of the proposed stacking ensemble models and the CNN models designed to be used at the first level. The learning curves of deep learning models are given in Figure 4. A learning curve can be derived that networks are overfitting, underfitting, or adapting as they should be during the training phase. At the beginning of the training phase, the network sees several training samples and the entire validation set, so it generalizes by rote. There is overfitting if the training accuracy is consistently below the validation accuracy after a specific period value. The fact that the number of training samples is greater than the number of validation samples causes a generalization gap. There is underfitting if the generalization gap is prominent in a learning curve. Since stacking models are hybrid models with two levels, a single learning curve cannot be drawn. However, it is crucial to interpret the learning curves of base learners, as the performance of base learners trained with endoscopic images are the main predictors that will affect the performance of stacking models.

## 4. Results 

**Figure 5:**
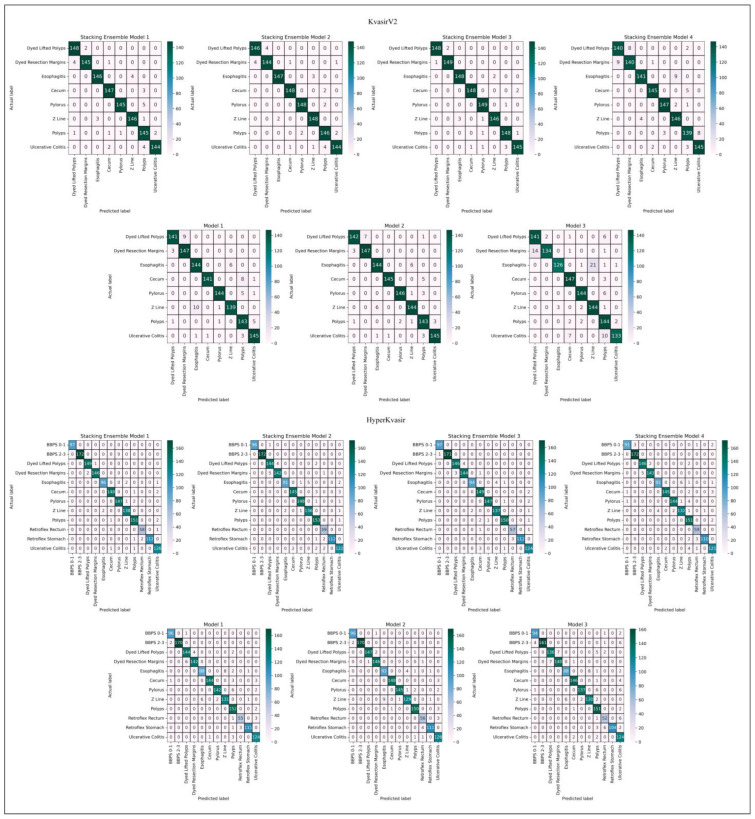
Confusion matrices.

In order to measure the performance of the established models, a test phase was carried out on separate test data. Performance measurement methods and explanations are given in Table 3. The confusion matrix is a basis for calculating performance measures that describe the full performance of the model by comparing the number of predicted labels with the actual labels. Confusion matrices represent true positive (TP), true negative (TN), false positive (FP), and false negative (FN) values. The class with the actual label is accepted as the positive class, and the cases where the actual label and the predicted label are the same give the TP value. In Figure 5, the confusion matrices of the models are displayed. When the confusion matrices of KvasirV2 and HyperKvasir datasets are examined, it is predicted that the stacking ensemble models give the highest classification performances. Stacking Ensemble Model 3 designed with LSVM meta-learner in KvasirV2 correctly predicted 1181 of 1200 test data. Stacking Ensemble Model 1 designed with MLP meta-learner in HyperKvasir correctly predicted 1540 of 1563 test data. In both datasets, Model 3 was the classifier with the most incorrect predictions (87 incorrect predictions for KvasirV2, 100 incorrect predictions for HyperKvasir).

Measurements obtained from the confusion matrix do not give precise information about the performance of the models but are used to calculate performance metrics. The test results calculated according to the selected metrics are given in Table 4. Stacking Ensemble Model 3, designed with LSVM meta-learner in the KvasirV2 dataset, and Stacking Ensemble Model 1, designed with MLP meta-learner in the HyperKvasir dataset, showed the highest performance. In the KvasirV2 dataset, Stacking Ensemble Model 3 reached 98.42%, 98.42%, 99.84%, 96.89%, and 98.19% in ACC, $F_{1}$ score, AUC, JSC, and MCC metrics, respectively. In the HyperKvasir dataset, Stacking Ensemble Model 1 reached 98.53%, 98.46%, 99.95%, 96.98%, and 98.39% values in ACC, $F_{1}$ score, AUC, JSC, and MCC metrics, respectively. Model 3, one of the base learners of the stacking models, a CNN model, showed the lowest performance in both datasets. In Model 3 of the KvasirV2 dataset, ACC, $F_{1}$ score, AUC, JSC, and MCC metrics reached 92.75%, 92.77%, 99.60%, 86.59%, and 91.77%, and in the HyperKvasir dataset, ACC, $F_{1}$ score, AUC, JSC, and MCC metrics reached 93.60%, 93.20%, 99.64%, 87.44%, and 93.02%.

The fact that the ACC, precision, recall, and $F_{1}$ score metrics in the KvasirV2 dataset have almost the same value is due to the balanced sample distribution. When the test results are examined, high precision and recall values mean that Type I and Type II errors are low. This result is of great importance for medical diagnostic applications. Stacking ensemble models are the classifiers that achieve the highest performance in both datasets. This factor proves that the proposed approach has a performance-enhancing effect on deep learning models. However, it is observed that the performance of Stacking Ensemble Model 4, designed using KNN as a meta-learner, is relatively low compared to the other three stacking models. Using metrics to compare the performance of algorithms is misleading. Performing a statistical test for performance benchmarking provides scientific evidence for results.

For reliable performance measurement, McNemar’s test [51], a variant of the $\chi^{2}$ test, was applied. The first step of McNemar’s test is to create the contingency table that gives the successful and unsuccessful predictions of the two selected algorithms. According to the contingency table, if b > c, algorithm A is more successful than algorithm B (b: number of predictions where algorithm A succeeds and algorithm B fails, c: number of predictions where algorithm B succeeds and algorithm A fails). In the second step, the z score is calculated to measure how different the two algorithms are from each other. If the z score is 0, the $H_{0}$ hypothesis, which argues that there is no significant difference between the two algorithms, is accepted. If the z score moves away from 0 to the positive direction, the $H_{1}$ hypothesis, which argues that there is a significant difference in performance between the two algorithms, is accepted.

Table 5 presents the results of McNemar’s test. The arrow symbols used in Table 5 show which algorithm is more successful. According to McNemar’s test, Stacking Ensemble Model 3 has the most successful classification performance on the KvasirV2 dataset. Although there is a performance difference between Stacking Ensemble Model 3 and Stacking Ensemble Model 2, it is not significant. The same is true for Stacking Ensemble Model 1 and Stacking Ensemble Model 2. Stacking Ensemble Model 1 is the algorithm with the best classification performance on the HyperKvasir dataset, and there is a significant difference with all other algorithms.

Stacking Ensemble Model 3 for the KvasirV2 dataset and Stacking Ensemble Model 1 for HyperKvasir are significantly more successful than deep learning models with a confidence level of over 99.50%. This result proves that the proposed approach improves the performance of deep learning models by a significant margin. Model 3 is significantly less successful for both datasets than all other algorithms. The lowest performance of Model 3 does not affect the stacking models because each base learner contributes the same amount to the ensemble prediction. The difference between deep learning models is the number of MLP blocks in the classifier layers. Model 2 performed best among deep learning models with a significant difference compared to Models 1 and 3. This indicates that increasing or decreasing the number of MLP blocks does not affect the performance of deep learning models. The number of MLP blocks should be considered a separate hyperparameter.

The McNemar’s test results mentioned so far support the performance metrics results. However, there are different results revealed by McNemar’s test. The Stacking Ensemble Model 4 and Model 1 have similar performance results, and the $H_{0}$ cannot be rejected. Model 2 Stacking Ensemble is more successful than Model 4, but the results are insignificant. The same is true between Model 2 and Stacking Ensemble Model 2 for the HyperKvasir dataset. If a base learner performs the same or better than a stacking model, the base learner should be preferred because the model has less complexity. In such a case, using stacking models extends training and maintenance times. Stacking models are designed to improve the performance of deep learning models, but not every stacking model is guaranteed to result in an improvement.

The stacking models presented in the application differ because the meta-learners are different machine learning algorithms. The failure of Stacking Ensemble Model 4, according to deep learning models, is due to the failure of the KNN algorithm, which is a meta-learner. In the same direction, the success of Stacking Ensemble Model 3 on the balanced dataset is due to MLP, and the success of Stacking Ensemble Model 1 on the unbalanced dataset is due to LSVM. Selected meta-learners determined the performance of the stacking models. 

Table 6 summarizes the performance difference over KvasirV2 and HyperKvasir datasets, where metrics and McNemar’s test show a performance rating of “+”. When Table 6 is examined, it is seen that McNemar’s test supports the results obtained from other measurement methods, except for Stacking Ensemble Model 4 and Model 1. For the KvasirV2 dataset, McNemar’s test shows no significant performance difference between Stacking Ensemble Model 4 and Model 1, while performance metrics show Model 1 outperforms Stacking Ensemble Model 4. Although the order of success for the ROC-AUC metric is different from McNemar’s test and other metrics, it is observed that the order of the best-performing models does not change. The lowest-performing is Stacking Ensemble Model 4 according to the ROC-AUC metric, while Model 3 is according to McNemar’s test and other metrics. The fact that the ROC-AUC metric offers different performance results draws attention to the importance of evaluating the results of artificial intelligence studies by applying statistical tests.

## 5. Discussion

The limitation of the proposed stacking models is that they add additional complexity to the already high-complexity deep learning models. High model complexity reduces interpretability and increases training and maintenance costs in terms of both hardware needs and computation time. The number of hyperparameters has a significant influence on model complexity. The high number of hyperparameters in the proposed approach made it difficult to search for distributed hyperparameters. Using an optimization algorithm instead of distributed hyperparameter search in tuning hyperparameters can increase performance and reduce model complexity and computation time. Another factor in keeping model complexity low is choosing simple but high-performance models with few parameters for base learners. Despite this disadvantage, a significant advantage that supports the recommendation of stacking models in this study is the performance-enhancing effects. Considering the use in real life, the testing phase takes seconds to be used in the field after the model is trained on the training data. For this reason, performance is more important than complexity in the models created for artificial intelligence applications in health. A proposed model should be reliable, objective, and predictable with high accuracy. 

Table 7 summarizes the state-of-the-art methods and results for GI tract findings classification. Although the differences and innovations of this study from the literature are clearly stated in Section 2, Table 7 presents the comparative results of the state-of-the-art methods and the proposed approach. The highest ACC value for the KvasirV2 dataset is 98.30%, reached with the transfer learning approach [23], while 97.99% is reached with the multi-feature fusion method for the HyperKvasir dataset [29]. With the approach proposed in this study, the highest classification ACC is 98.42% for the KvasirV2 dataset and 98.53% for the HyperKvasir dataset. In general, although state-of-the-art methods show low performance on the HyperKvasir dataset, the high performance of this study is remarkable. It is seen in Table 7 that the performance of the proposed approach is higher than the other state-of-the-art methods proposed in the literature.

Based on findings such as the rapidity and high performance of the proposed approach and the statistical proof of its high performance, the methods presented in this study can be applied in the clinical environment. The proposed approach could enable the development of new intelligent endoscopic devices, supporting early life-saving diagnosis with objective and reliable predictions. This study can reduce specialists’ workload, save cost and time, and help prevent misdiagnosis and treatment caused by specialist errors. In addition to the clinical benefits, the proposed approach can be applied to other topics studied in the field of artificial intelligence in medicine and may provide new high-performance solutions.

## 6. Conclusions

In this study, innovative and hybrid stacking ensemble models, in which base learners are deep learning algorithms, and meta-learners are machine learning algorithms, are proposed for GI tract classification. The results of the selected metrics for performance evaluation were supported by applying McNemar’s test. According to the experimental studies conducted on two separate datasets, balanced and unbalanced, stacking models performed approximately 99% with a significant difference. The proposed stacking approach increases the performance of deep learning models and outperforms the state-of-the-art studies in the literature. This study provides an artificial intelligence system that will produce high-performance, reliable, objective, and fast results when applied in the clinical environment, helping endoscopy specialists in many factors and providing an early diagnosis.

## Figures and Tables

**Table 1:** Configuration of new deep learning models.

Model 1	Model 2	Model 3
14 weight layers	15 weight layers	16 weight layers
input (150, 150, 3)		
3 × 3 conv—64 + gelu (150, 150, 64) 3 × 3 conv—64 + gelu (150, 150, 64)		
2 × 2 max-pooling, stride 2 (75, 75, 64)		
batch_normalization		
3 × 3 conv—128 + gelu (75, 75, 128) 3 × 3 conv—128+ gelu (75, 75, 128)		
2 × 2 max-pooling, stride 2 (37, 37, 128)		
batch_normalization		
3 × 3 conv—256 + gelu (37, 37, 256) 3 × 3 conv—256 + gelu (37, 37, 256) 3 × 3 conv—256 + gelu (37, 37, 256)		
2 × 2 max-pooling, stride 2 (18, 18, 256)		
batch_normalization		
3 × 3 conv—512 + gelu (18, 18, 512) 3 × 3 conv—512 + gelu (18, 18, 512) 3 × 3 conv—512 + gelu (18, 18, 512)		
2 × 2 max-pooling, stride 2 (9, 9, 512)		
batch_normalization		
3 × 3 conv—512 + gelu (9, 9, 512) 3 × 3 conv—512 + gelu (9, 9, 512) 3 × 3 conv—512 + gelu (9, 9, 512)		
2 × 2 max-pooling, stride 2 (4, 4, 512)		
global-average-pooling—512		
dense—1024 + gelu dropout—0.5 batch_normalization	dense—2048 + gelu dropout—0.5 batch_normalization	dense—4096 + gelu dropout—0.5 batch_normalization
dense—8/12 + softmax	dense—1024 + gelu dropout—0.5 batch_normalization	dense—2048 + gelu dropout—0.5 batch_normalization

	dense—8/12 + softmax	dense—1024 + gelu dropout—0.5 batch_normalization

		dense—8/12 + softmax

**Table 2:** The values of the hyperparameters.

Base Learners Hyperparameter	Value
Batch size	32
Learning rate	2 × 10^−3^
Epoch	50
Loss function	sparse_categorical_crossentropy
Optimization algorithm	Adam [48]
Meta-Learners Hyperparameter	Value
Logistic regression	
C	10
Solver	newton-cg [49]
max_iter	100
multi_class	ovr
Support vector machine	
C	0.1
Kernel	linear
Loss	squared_hinge
max_iter	100
multi_class	crammer_singer [50]
Multi-layer Perceptron	
hidden_layer_sizes	128, 64
Activation	ReLU
Solver	Adam
learning_rate_init	1 × 10^−3^
max_iter	100
K-nearest neighbors	
n_neighbors	15
*P*	1
Metric	manhattan_distance

**Table 3:** Performance metrics.

Metrics	Description
True Positive (TP)	A situation where the actual and predicted data point class is correct.
True Negative (TN)	A situation where the actual and predicted data point class is incorrect.
False Positive (FP)	A situation where the actual data point class is false and the predicted data point class is true.
False Negative (FN)	A situation where the actual data point class is true and the predicted data point class is false.
ROC-AUC	The AUC (area under the ROC curve) value measures how well two classes can be distinguished, and a value of 1 is desired for perfect discrimination.
Accuracy (ACC)	It is the number of correct guesses divided by the total number of guesses and is required to be 1 for perfect discrimination. $\frac{TP+TN}{TP+FP+FN+TN}$
Precision	A Type I error occurs when a true null hypothesis ($H_{0}$) is rejected. A Type I error is the definition of a class as normal that would be sick. That is why the precision metric is critical in medical applications. $\frac{TP}{TP+FP}$
Recall	A Type II error occurs when a false null hypothesis ($H_{0}$) is accepted. Type II error is when a class that would be defined as normal is defined as sick. $\frac{TP}{TP+FN}$
$F_{1}$ Score	It is the harmonic mean of precision and recall and is required to be 1 for perfect discrimination. It does not explicitly report whether a model has Type I or Type II errors but provides an objective measure of unbalanced classification problems. $2\times \frac{Precision\times Recall}{Precision+Recall}$
Jaccard similarity coefficient (JSC)	It is the ratio of the intersection of two sets of labels, actual and predicted, to the union and is required to be 1 for perfect discrimination. $\frac{TP}{TP+FN+FP}$
Matthews correlation coefficient (MCC)	In the case of multiple classification, the minimum value is between −1 and 0, and the maximum value is +1. The $F_{1}$ and ACC metrics are positive class-dependent, but MCC produces positive and negative class-dependent results. Many studies consider it the most reliable metric in unbalanced classification problems. $\frac{TP\times TN-FP\times FN}{\sqrt{\left(TP+FP\right)\left(TP+FN\right)\left(TN+FP\right)\left(TN+FN\right)}}$

**Table 4:** Test results.

Classifier	ACC (%)	Precision (%)	Recall (%)	$F_{1}$ Score (%)	ROC-AUC (%)	JSC (%)	MCC (%)
KvasirV2							
Model 1	95.33	95.44	95.33	95.35	99.69	91.15	94.68
Model 2	96.33	96.38	96.33	96.34	99.73	92.98	95.81
Model 3	92.75	93.21	92.75	92.77	99.60	86.59	91.77
Stacking Ensemble Model 1	97.17	97.22	97.17	97.18	99.82	94.54	96.77
Stacking Ensemble Model 2	97.58	97.59	97.58	97.58	99.80	95.30	97.24
Stacking Ensemble Model 3	**98.42**	**98.42**	**98.42**	**98.42**	**99.84**	**96.89**	**98.19**
Stacking Ensemble Model 4	95.25	95.28	95.25	95.25	98.87	90.98	94.57
HyperKvasir							
Model 1	95.91	95.87	95.63	95.71	99.79	91.86	95.51
Model 2	97.06	96.55	96.87	96.69	99.86	93.69	96.77
Model 3	93.60	93.43	93.25	93.20	99.64	87.44	92.99
Stacking Ensemble Model 1	**98.53**	**98.45**	**98.48**	**98.46**	**99.95**	**96.98**	**98.39**
Stacking Ensemble Model 2	97.25	97.11	97.23	97.14	99.83	94.49	96.98
Stacking Ensemble Model 3	97.76	97.66	97.65	97.65	99.90	95.43	97.54
Stacking Ensemble Model 4	96.29	96.19	96.19	96.15	98.62	92.63	95.93

**Table 5:** Results of McNemar’s test.

KvasirV2						
	Model 2	Model 3	SEM 1	SEM 2	SEM 3	SEM 4
**Model 1**	↑1.33	←2.82	↑2.71	↑2.82	↑4.16	0
**Model 2**		←4.22	↑1.27	↑1.66	↑3.07	←1.23
**Model 3**			↑5.45	↑5.39	↑6.57	↑2.49
**SEM 1**				↑0.51	↑1.92	←2.33
**SEM 2**					↑1.42	←3.74
**SEM 3**						←4.62
**HyperKvasir**						
	**Model 2**	**Model 3**	**SEM 1**	**SEM 2**	**SEM 3**	**SEM 4**
**Model 1**	↑1.88	←3.4	↑5.04	↑2.52	↑3.37	↑0.58
**Model 2**		←5.3	↑3.02	↑0.25	↑1.35	←1.26
**Model 3**			↑7.97	↑5.94	↑6.57	↑4.1
**SEM 1**				←2.93	←1.7	←4.86
**SEM 2**					↑1.01	←1.96
**SEM 3**						←2.77

**Table 6:** Summary of the performances.

	Model 1	Model 2	Model 3	SEM 1	SEM 2	SEM 3	SEM 4
McNemar’ s Test							
KvasirV2	++	+++	+	++++	+++++	++++++	++
HyperKvasir	++	++++	+	+++++++	+++++	++++++	+++
ROC-AUC							
KvasirV2	+++	++++	++	++++++	+++++	+++++++	+
HyperKvasir	+++	+++++	++	+++++++	++++	++++++	+
ACC, $F_{1}$, JSC, and MCC							
KvasirV2	+++	++++	+	+++++	++++++	+++++++	++
HyperKvasir	++	++++	+	+++++++	+++++	++++++	+++

**Table 7:** Overview of the literature.

Author, Year, Reference	Approach	Results (ACC %)
	KvasirV2	
Öztürk and Özkaya, 2020 [26]	LSTM based CNN	97.90
Öztürk and Özkaya, 2021 [27]	Residual LSTM layered CNN	98.05
Hmoud Al-Adhaileh et al., 2021 [24]	Transfer learning	97.00
Mohapatra et al., 2021 [32]	2D-DWT and CNN	97.25
Khan et al., 2022 [30]	Bayesian optimal deep learning feature selection	98.02
Yogapriya et al., 2021 [25]	Transfer learning	96.33
Afriyie et al., 2022 [34]	Dn-CapsNet	94.16
Khan et al., 2022 [31]	Moth-Crow Optimization with DCCA Fusion	97.20
Dheir and Abu-Naser, 2022 [23]	Transfer learning	98.30
	**HyperKvasir**	
Ramamurthy et al., 2022 [29]	Multi-feature fusion method	97.99
Mohapatra et al., 2022 [33]	EWT and CNN	96.65
Dutta et al., 2021 [28]	Tiny Darknet	75.80 (MCC)
Borgli et al., 2020 [22]	ResNet-152 + DenseNet-161	90.20 (MCC)
	**KvasirV2 + HyperKvasir**	
Wang et al., 2022 [35]	Convolutional-capsule network	KvasirV2 94.83; HyperKvasir 85.99
This study	Deep learning-based hybrid stacking ensemble models	KvasirV2 98.42; HyperKvasir 98.53

## Data Availability

Data are contained within the article. The data presented in this study are available in [21,22].
